# Supplementary material for: Minimizing carry-over PCR contamination in expanded CAG/CTG repeat instability applications
Source: Sci Rep. 2017 Dec 21;7:18026. doi: 10.1038/s41598-017-18168-2 (PMC5740160; doi:10.1038/s41598-017-18168-2)
Supplement: Supplementary file 1 — Supplementary information [file 41598_2017_18168_MOESM1_ESM.pdf]

Minimizing carry-over PCR contamination in expanded CAG/CTG repeat instability applications

Lorène Aeschbach and Vincent Dion

Supplemental Material and Methods

Center for Integrative Genomics
Faculty of Biology and Medicine
University of Lausanne
1015 Lausanne
Switzerland

Corresponding author: vincent.dion@unil.ch

Small Pool PCR for expanded CAG/CTG repeats
using Ung-pre-treatment and uracil-containing amplicons
Last update: 2017-09-19

Goal: Small-pool PCR is the gold standard for measuring quantitatively the frequency of expanded repeats. This protocol takes you through the process and includes extra steps to prevent carry-over contamination. Specifically, it uses uracil-containing amplicons that can be degraded by the bacterial uracil *N*-glycosylase, Ung, which is used prior to the PCR. This protocol outlines the materials and methods used to perform SP-PCR at the GFP transgene contained in GFP(CAG)x cells[1]. You will need to adjust the primers, PCR program, probe, and/or hybridization temperature.

Note: To minimize any other potential sources of carry-over contamination, we suggest that you work under a hood that can be decontaminated with UV. Ideally this hood is located in a different room from the one in which the PCRs are analyzed and manipulated further. In addition, we strongly advise that you use pipets and solutions dedicated to SP-PCR.

Materials and solutions needed

- α-^{32}P-dCTP (Perkin Elmer #BLU013H250UC)
- Agarose LE (Promega #V3125)
- Alkaline transfer buffer (0.4M NaOH, 1M NaCl)
- dCTP, dATP, and dGTP at 100mM each (LabGene #KK1007). Make a 10mM each stock.
- DMSO
- DNA Probe for the locus of interest amplified with dUTP.
- dUTP 100mM (Thermo Fisher Scientific #R0133). Make a 20mM stock.
- Fish sperm DNA 10mg/ml (Roche #11467140001)
- Heat labile Uracil-DNA *N*-Glycosylase (Roche #11775367001)
- Micro bio-spin 30 chromatography columns (BioRad #7326223)
- Molecular weight marker 100bp (Promega #G8291)
- Neutralization buffer (1.5M NaCl, 0.5M Tris base pH7.4)
- Nick Translation kit (Thermo Fisher Scientific #18160-010)
- Nuclease and DNA-free water
- oVIN-460 (5’- TCTGCAAATTCAGTGATGC) - 10µM
- oVIN-1425 (5’- GACCTCATACGAAGATAGGCTT) - 10µM
- PeqGreen DNA/RNA dye (PeqLab #37-5010)
- Phusion U Green Hot Start DNA Polymerase kit (Thermo Fisher Scientific #F-556L)
- Proteinase K 2mg/ml (Promega #V3021)
- 20X SSC (3M NaCl, 0.3M NaCitrate pH7.0)
- 1X TAE (40mM Tris-acetate / 1mM EDTA) For 1L of 10X = 48.4g Tris, 11.42ml glacial acetic acid, 37.2g $Na_2EDTA*2H_2O$
- Ultrahyb buffer (Thermo Fisher Scientific #AM8670)
- Wash buffer (0.5X SSC, 0.1% SDS)
- Fluorescent ruler
- Hybridization oven + cylinders
- Large gel migration system (20 cm x 25 cm) with recirculation (e.g. Owl A5)
- Nylon membrane (Fisher Scientific #NP0HYA0010)
- Parafilm

- Whatman filter paper (Macherey Nagel #742113)

Step 1: Genomic DNA dilutions and PCR amplification

Establishing the concentration of DNA is often error-prone. Thus, to determine the concentration of amplifiable genomes in your sample, the first step is to perform a serial dilution. This is discussed in detail in[2] (it is a great paper on this method, go read it). Once you have determined the appropriate concentration for each sample, then you can go back and run multiple membranes with that concentration. When doing the dilutions, it is advisable not dilute more than 10 fold at a time, and not to pipet volumes smaller than 10µl. In addition, vortexing increases the chances of carry-over contamination – mix by tapping the tube gently. First try dilutions of 100pg/µl, 50pg/µl, 25pg/µl, and 12.5pg/µl. Set up 7 reactions plus one negative control without DNA (but that has undergone the dilutions) for each concentration. For each set of PCRs, add one positive control with 10ng of DNA to be run on a small gel to make sure that the reaction has worked (you will not see a product on the gel for your dilutions).

PCR Master Mix:

For 10µl reaction (final concentration in brackets):

- Buffer 5X: 2µl (1X)
- dNTP mix without dTTP: 0.2µl (0.2mM)
- dUTP: 0.2µl (0.4mM)
- oVIN-460: 0.5µl (0.5µM)
- oVIN-1425: 0.5µl (0.5µM)
- DMSO: 0.3µl (3%)
- UNG: 0.1µl (0.1U)
- Phusion Taq: 0.2µl (0.4U)
- DNA: 1µl of the appropriate concentration
- H_2O: 5µl

PCR program:

20°C	10' (For hlUng activity)	
95°C	2'(To inactive hlUng)	
95°C	5'	
95°C	30''	35X
60°C	30''	
72°C	1'30''	
72°C	10'	
4°C	2'	

Inactivate hlUng

Add 0.5µl of 2mg/ml Proteinase K to have a final concentration of 100µg/ml. Incubate 1 hour at 37°C.

Step 2: Gel migration

- Make a 2% agarose gel in 1X TAE (350ml) adding 3.5µl of PeqGreen DNA dye.
- The gel electrophoresis chamber holds 2L of buffer (of an Owl A5).
- Run the gel at 180V for 5 hours in 1X TAE – the time and voltage depend on your power source and gel system.

- After the run, remove the unnecessary parts of the gel and take a picture with the fluorescent ruler to be able to determine the size of the bands obtained on the membrane at the end.

Step 3: Transfer

- Incubate the gel twice for 20min in alkaline transfer buffer with light shaking.
- Fill both ends of a transfer apparatus with alkaline transfer buffer.
- Cut a piece of Whatman paper wide enough to cover the surface where your gel will sit and long enough so that it dips into the alkaline transfer buffer at both end. Pour some more alkaline transfer buffer on top so that the filter paper is wet.
- Place your gel onto the filter paper wells down.
- Make sure there is no bubble between the filter paper and the gel. We use a 5ml plastic pipette to roll out the bubbles.
- Cut a piece of membrane exactly the size of your gel.
- Cut a corner so that you remember the orientation and what face of the membrane you DNA will be.
- Place the dry membrane on top of the gel.
- Make sure there is no bubble between the gel and the membrane.
- Cut two pieces of filter paper the size of your membrane, dip each one at a time in alkaline transfer buffer and stack them top of the membrane. Make sure there is no bubble in between them.
- Every bit of filter paper not touching the gel should be covered with parafilm so as not to short circuit the transfer.
- Add plenty of paper towel on top.
- Put a Plexiglas plate on top and a paper weight (not too heavy) to distribute the load around.
- Transfer over night. Make sure there is enough of the alkaline transfer buffer – the transfer will stop if you run out.
- Wash the membrane with Neutralization buffer for 5 min. You may stop here until you are ready to proceed. Store membrane in the neutralization buffer at 4°C.

Step 4: Labeling the probe

The probe DNA should contain the region amplified. In our case, we generate the probe using the Ung-pretreatment and dUTP-containing PCR as in Aeschbach and Dion 2017 using oVIN-1425 and oVIN-460 as primers and pVIN-109 as template. This plasmid contains 40 CAGs and the GFP reporter construct. Work in an approved radioactivity room.

- Set up the nick translation reaction using the solutions from the nick translation kit:
    - Use 50ng to 200ng of probe DNA.
    - 5µl dNTP mix without dCTP (from the nick translation kit)
    - 5µl of the enzyme mix (from the kit)
    - 5µl Alpha-^{32}P-dCTP
    - Adjust to 50µl with H_2O
- Incubate 1 hour at 16°C.
- Add 5 µl of stop buffer
- Purify the labelled DNA from unincorporated dNTPs using the Micro bio-spin 30 chromatography columns:

- Spin out the buffer at 1000g for 1 min, discard the flow-through.
- Add your labeling reaction to the column gently in the middle of the beads (if you load on the side the column will not be efficient.
- Spin 1 min at 1000g and use the flow-through.

Up to three membranes can be probed with one nick translation reaction.

Step 5: Hybridization:

- Warm up 15ml of Ultrahyb buffer to 42°C in the hybridization cylinder.
- Place the membrane in the hybridization cylinder with the DNA side facing inwards.
- From this point on it is critical to keep the membrane wet and at warm temperature. If the membrane dries, the resulting blot will have blotches and will not be of publication quality.
- Remove all bubbles that may remain between the membrane and the cylinder.
- Boil 500µl of salmon sperm DNA for 10min. Put on ice, then add in the Ultrahyb solution.
- Pre-hybridize for at least 1hr at 42°C.
- Boil the probe for 5 minutes and add it immediately to the hybridization buffer.
- Incubate for 2 hours at 42°C.
- Pour out the hybridization buffer and add some pre-warmed wash buffer.
- Incubate for 30 minutes.
- Pour out the buffer, add fresh wash buffer and incubate for another 30 minutes.
- Take the membrane and put it inside of plastic sheet protectors.
- Put all inside the cassette with a phosphoscreen.
- Expose at room temperature overnight.
- Use a Typhoon to reveal the blot.

Step 6: Analysis of results

To determine the appropriate concentration to use for a specific sample, look for the dilution yielding 1 empty lane out of the 7. According to a Poisson distribution, this corresponds to about 2 genome/lane. Then you can work out with your dilutions what concentration you need to get the desired number of alleles. You can then set up a second blot (or more) with the appropriate concentration and the number of repeats above and below the mean can be calculated. The sizing is done on the resulting blot using the picture with the ruler taken before the transfer.

References

1. Cinesi, C., Aeschbach, L., Yang, B. & Dion, V. Contracting CAG/CTG repeats using the CRISPR-Cas9 nickase. *Nat Commun* **7**, 13272 (2016).
2. Gomes-Pereira, M., Bidichandani, S.I. & Monckton, D.G. Analysis of unstable triplet repeats using small-pool polymerase chain reaction. *Methods Mol Biol* **277**, 61-76 (2004).

Unaltered gel pictures for Fig. 1

A.

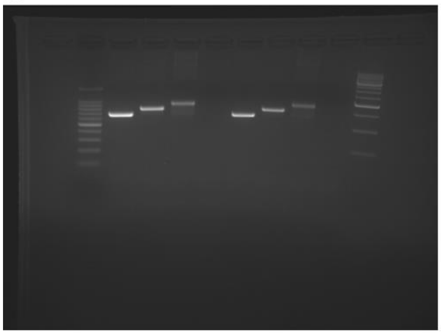

B.

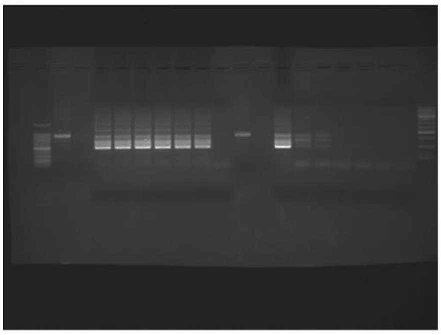

Unaltered gel picture for Fig. 2

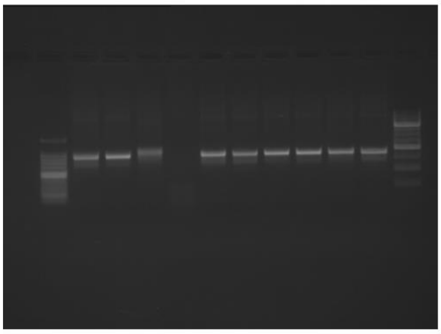

Unaltered gel picture for Fig. 3

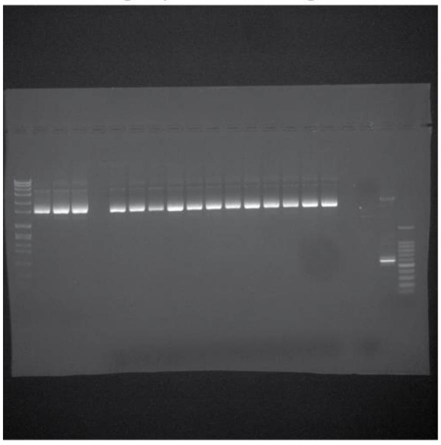

Uncropped blots for figure 5:

A. Ung + Proteinase K

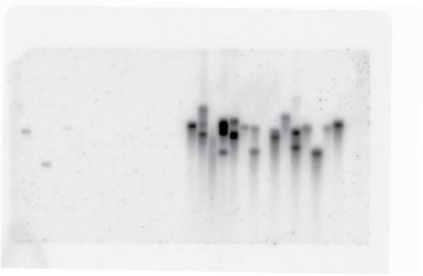

B. hlUng + Proteinase K

Cas9 nickase + pPN10 + dTTP

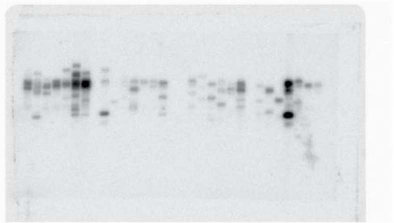

Cas9 nickase + pPN10 + dUTP

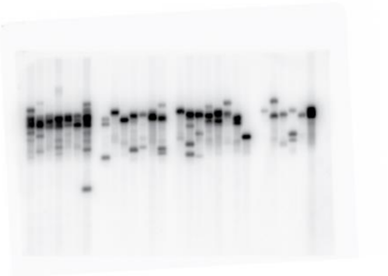

Cas9 nickase + sgCTG+ dTTP

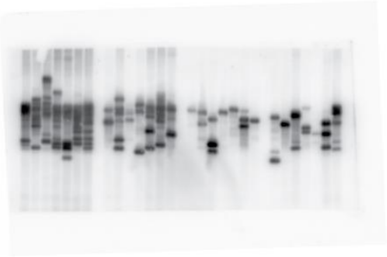

Cas9 nickase + sgCTG+ dUTP

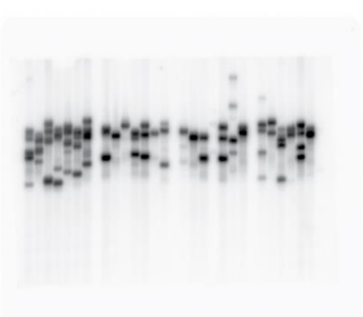

Uncropped blots for figure 6:

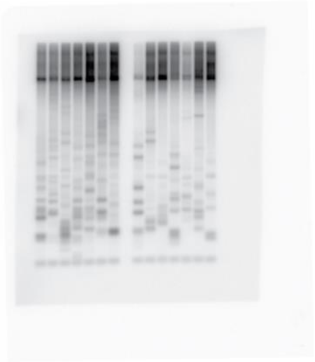

Suppl. Fig. 1:
Unaltered gels and blots for Figures 1-3 and 5-6.
